# The Importance of Peer Reviewing

**DOI:** 10.1100/tsw.2000.18

**Published:** 2001-04-04

**Authors:** Jack Franklin

## Abstract

The importance of peer reviewing articles submitted to scientific journals is a subject that conferences and journals regularly revisit. In general such visits are brief. Although a few horror stories abound: competitor holds up acceptance of paper to submit his own and so get priority or competitor pours sufficient scorn on a good paper to darken its worth.

Most people agree that scientific articles have to be refereed at least because refereeing helps an author prepare a better paper.

Jayne Marks, Publishing Director of *Nature*, discussed the added value this process gives to science at last week's **STM Publishing Conference** organised by PIRA in London, UK. Ms. Marks re-emphasised the differences in refereeing, from *Nature*'s internal editors through to the *BMJ*'s 'open refereeing' where the referees are named. She explained that referees 'add value' to the publishing process by guaranteeing the name and image of a title AND by acting as unpaid tutors to authors who require comment and correction to get their papers ready for publication – somewhere at least!

While no one can argue against the added value a referee can provide in improving a paper, the real reason for refereeing is surely to ensure that a paper fits the aims and scope of the journal and represents good science. Ms. Marks confided that often this is too rigidly enforced, and that Watson and Crick's famous DNA paper would probably not have been accepted by today's *Nature*. It might actually not have been accepted by many a 'top journal' and so would have had to dribble down the column until a lesser journal needed the pages. Unfortunately, there appears to be fewer and fewer editors willing or able to take a risk really challenging papers. Today, refereeing still has to ensure that the material is 'scientifically correct' but probably, more importantly, it has to be relevant to the subscriber, who pays in advance for a journal believing it will contain relevant articles of interest and quality.

Yet, even given this restricted aim, and despite all the claims, refereeing is not an exact science. Sadly, the referee is not always really in tune with the paper before him and in many cases may not be as well placed in the field as the author. Neither of these points is a reason for saying a good critical scientist cannot referee a paper, but it leaves the system open to criticism. Furthermore, increasingly, referees are also not given sufficient information to judge the work, as all too often conclusions will rely upon the analysis of data that continue to reside in the author's database. Such data are perhaps inaccessible, or cannot be handled without the sophisticated software tools available to the author, leaving the referee unable to do more than 'assume' or extrapolate. There is little doubt that this fact has seriously hampered reviewers in fields like crystallography or molecular genetics.

Moreover, even when the paper contains everything necessary for a decision to be made, and when the reviewing expertise is in tune, referees often fail to agree. Many journals have to use a third reviewer when the initial process ends up with conflicting opinions. Twenty years ago, the editorial board of a major and highly respected journal had to be persuaded not to promote the fact that 'each paper was seen by 2.8 referees' after it was pointed out that this meant that of every 100 manuscripts sent out to the standard two referees, 80 needed to be sent to a third referee for a final 'decision'. Perhaps this was (and still is) an indication of what some critics of refereeing claim is an intentioned abuse, i.e., that the safety-gate of refereeing can actually make an author lazy. It is far easier/safer to submit a manuscript to an anonymous reviewer who might point out that the mathematical formula on a particular page is inaccurate, than to find the magnitude of the mistake on publication!

Above all other considerations, the worthiness of refereeing comes down to whether or not the article is accurate and can be trusted. Often the end-reader makes the final and conclusive decision. The web might now be offering a useful alternative. At the same meeting, Bill Town, Director of Operations at Chemweb, reported on the early launch of a chemistry pre-print service run by his service. While this service is still very young, a number of scientists have submitted papers to his site so that they can actually be openly refereed, i.e., they are immediately open to public criticism. As Dr. Town reported, much has to be done before this service equals the accepted route of pre-print-published articles in physics, but a new route for chemists has been opened. Authors can submit their papers, which are reviewed for 'chemical correctness', before being mounted on the Chemweb site. Comments and alterations can be added and the author is then free to submit the article to a traditional

Previously posted in NewsLab, TheScientificWorld.
© 2000 with author.

journal when he thinks he can appropriately do so. Chemweb will even help in that process and while this might - despite claims of being publisher neutral - benefit the parent company, Elsevier, it will certainly help hard-pressed authors as well.

The initial take-up has started and a number of papers are being handled at this moment. The aim is, at least, to start by living alongside the traditional journals, as happens in the physics world, but is this the beginning of a division in the publishing world? There is still a clear anomaly that a paper that has been public for some time, on the transparent world-wide-web, somehow only gains real credence when it is accepted by a traditional journal. This obviously says more about the established system of gaining 'brownie points through citation analysis' than in the final refereeing preprinted papers will receive. Chemweb is testing the market whether it admits it or not.

The end game might be some way away, but pre-printing and web-based criticism will offer authors a new regime. A paper can be previewed until such time as the author decides to 'set it in stone'. An e-paper is soon going to be easier to reach than a paper-paper. While present pre-print services are run on the efforts of the communities they support, they are cheaper to run than established journals. If citation ratings could be devised for pre-prints, as they can, the desired impact of an article can be measured. With the web offering authors the chance of reaching 'anyone who's interested', the time might come when the referee is replaced by the general community. Just like it used to be with society publications!

Jack Franklin
asfra

.
